# Impact of four-dimensional cone-beam computed tomography on target localization for gastric mucosa-associated lymphoid tissue lymphoma radiotherapy: reducing planning target volume

**DOI:** 10.1186/s13014-020-01734-w

**Published:** 2021-01-14

**Authors:** Yoshinobu Shimohigashi, Ryo Toya, Tetsuo Saito, Yumiko Kono, Yasuhiro Doi, Yoshiyuki Fukugawa, Takahiro Watakabe, Tadashi Matsumoto, Yudai Kai, Masato Maruyama, Natsuo Oya

**Affiliations:** 1 Department of Radiological Technology, Kumamoto University Hospital, 1-1-1 Honjo, Chuo-ku, Kumamoto, 860-8556 Japan; 2 Department of Radiation Oncology, Kumamoto University Hospital, Kumamoto, Japan

**Keywords:** Malignant lymphoma, Mucosa-associated lymphoid tissue lymphoma, Four-dimensional computed tomography, Four-dimensional cone-beam computed tomography, Image-guided radiotherapy, Planning target volume

## Abstract

**Background:**

Radiotherapy of gastric mucosa-associated lymphoid tissue (MALT) lymphoma should be delivered to the entire stomach with planning target volume (PTV) that accounts for variations in stomach volume, respiratory movement, and patient set-up error. In this study, we evaluated whether the use of four-dimensional cone-beam computed tomography (4D-CBCT) reduces the PTV.

**Methods:**

Eight patients underwent radiotherapy with 15 fractions of gastric MALT lymphoma using 4D-CBCT. PTV structures of 5–30 mm margins (5 mm intervals) from the clinical target volume (CTV) delineated based on the 4D-CT images (CTV-4D) were generated. For the target localization, we performed matching based on skin marking (skin matching), bone anatomy (bone matching), and stomach anatomy (4D soft-tissue matching) based on registration between planning CT and 4D-CBCT images from 10 phases. For each patient, we calculated the covering ratio (CR) of the stomach with variable PTV structures, based on the 4D-CBCT images, with a total of 150 phases [CR (%) = (number of covering phases/150 phases) × 100], for three target localization methods. We compared the CR values of the different target localization methods and defined the PTV with an average CR of ≥ 95% for all patients.

**Results:**

The average CR for all patients increased from 17.9 to 100%, 19.6 to 99.8%, and 33.8 to 100%, in the skin, bone, and 4D soft-tissue matchings, respectively, as the PTV structures increased from 5 to 30 mm. The CR obtained by 4D soft-tissue matching was superior to that obtained by skin (*P* = 0.013) and bone matching (*P* = 0.008) for a PTV structure of 15 mm margin. The PTV required an additional margin of 20 mm (average CR: 95.2%), 25 mm (average CR: 99.1%), and 15 mm (average CR: 98.0%) to CTV-4D for the skin, bone, and 4D soft-tissue matchings, respectively.

**Conclusions:**

This study demonstrates that the use of 4D-CBCT reduces the PTV when applying 4D soft-tissue matching, compared to skin and bone matchings. Additionally, bone matching does not reduce the PTV as compared with traditional skin matching.

## Background

Radiotherapy of gastric mucosa-associated lymphoid tissue (MALT) lymphoma provides excellent long-term local control and survival [1–4]. The clinical target volume (CTV) for gastric MALT lymphoma is defined as the entire stomach, and the planning target volume (PTV) is defined as CTV along with an additional margin, which accounts for variations in stomach volume, respiratory movement, and patient set-up error. Therefore, the target volume for gastric MALT lymphoma is very large. Moreover, it is well-known that intrafractional gastric motion and interfractional variation of the stomach volume occur during treatment for gastric lymphoma [5–8]. To address these issues, four-dimensional (4D) computed tomography (CT) is currently used to consider intrafractional gastric motion during treatment planning [9–11], and image-guided radiotherapy (IGRT) using daily CT images (CT-IGRT) is used to evaluate interfractional changes in stomach volume during the course of the treatment [8, 12]. Historically, before the era of IGRT, PTV was typically defined as CTV along with an approximately 20–30-mm margin with matching based on skin marks (skin matching) [1, 5, 6]. Even after the introduction of CT-IGRT, a CTV along with an approximately 20 mm margin was required with matching based on bone anatomy (bone matching) in the free breathing (FB) condition [7, 8]. Recently, using a breath-hold technique, Wang et al. reported that daily CT-IGRT with matching based on stomach anatomy (soft-tissue matching) enables excellent target coverage with a small additional margin of 5–10 mm [12]. However, the use of a breath-hold technique is not prevalent in all institutions.

4D cone-beam CT (4D-CBCT) has recently been introduced into the clinical setting and is used for IGRT of lung and abdominal tumors [13–16]. Furthermore, 4D-CBCT has been used for assessing both the intrafractional and interfractional movements of a tumor and the PTV settings [17, 18]. We previously reported a treatment method that employed IGRT using 4D-CBCT images (4D-CBCT-IGRT) for a patient with gastric MALT lymphoma, and we suggested that this approach provides more precise target localization [19]. However, our previous report did not systematically evaluate the impact of 4D-CBCT-IGRT on the target localization during the treatment course of gastric MALT lymphoma.

Intensity-modulated radiation therapy (IMRT) has recently been introduced for gastric lymphoma radiotherapy to obtain dose distributions that are highly conformal to the PTV while minimizing the dose to the organs at risk (OARs), such as the liver, spinal cord, and kidneys [20–23]. IMRT should be delivered with high accuracy in combination with precise IGRT. Hence, determining whether the use of 4D-CBCT-IGRT contributes to a reduction of PTV is important in minimizing the dose to the OARs in patients with gastric MALT lymphoma.

In this study, we evaluated whether the use of 4D-CBCT-IGRT reduced the PTV by determining the required PTV for three target localization methods, based on skin, bone, and 4D soft-tissue matching, for gastric MALT lymphoma radiotherapy in the FB condition.

## Materials and methods

### Patients

This retrospective study was approved by the institutional research ethics board of our hospital. Informed consent for treatment and the use of 4D-CBCT-IGRT and its images for this study was obtained from all patients. Eight patients who completed 4D-CBCT-IGRT for gastric MALT lymphoma radiotherapy at our hospital between May 2017 and October 2019 were included in this study.

### 4D-CT imaging and structure generation

All patients were instructed to fast for at least 8 h before planning CT simulation and treatment to minimize variations in stomach volume. They underwent CT simulation in the supine position with their arms raised; a LightSpeed RT (GE Healthcare, Chicago, IL) or a Discovery RT CT scanner (GE Healthcare) was used for the CT simulation. 4D-CT scans were performed using a real-time position management system (Varian Medical Systems, Palo Alto, CA) or smart deviceless 4D (GE Healthcare) [24]. The scan parameters were set to 120 kV, 70 mA, a gantry rotation time of 0.5–1.0 s, a slice thickness of 2.5 mm, and cine mode. The cine durations were set to the respiratory cycles plus the gantry rotation time. The cine images were sorted into 10 phases using a phase-binning algorithm. The average intensity projection (AIP) CT images were generated from the projection data of all phases. In cases where AIP CT images could not be generated, slow CT images were acquired in the axial mode, with a gantry rotation time of 4 s and a slice thickness of 2.5 mm [25, 26].

**Fig. 1:**
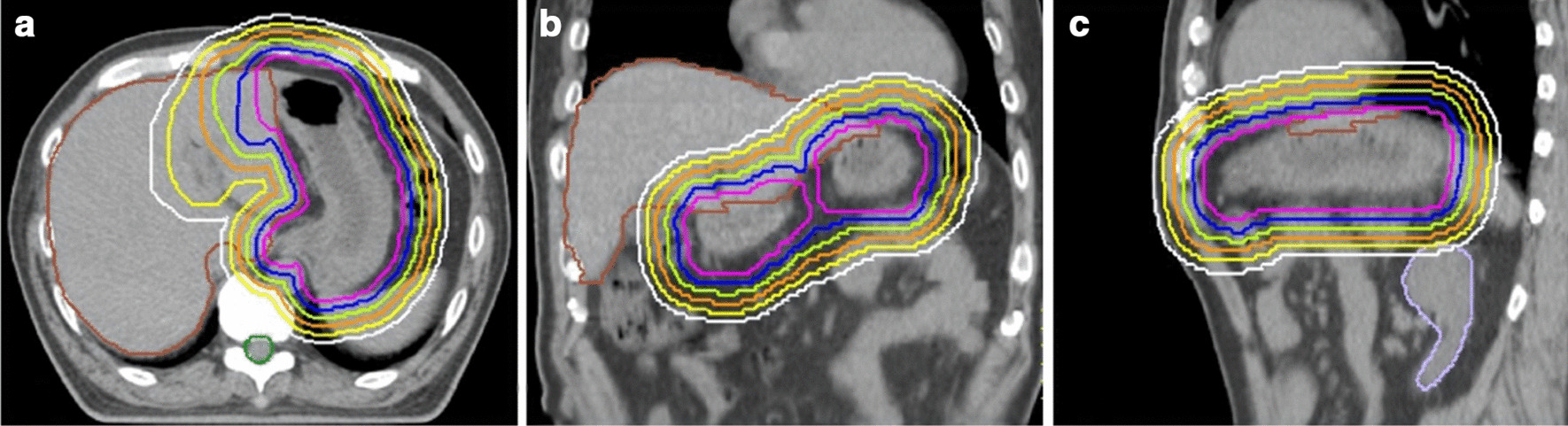
PTV structures for the retrospective evaluation. PTV structures with 5 mm (purple), 10 mm (blue), 15 mm (yellow-green), 20 mm (orange), 25 mm (yellow), and 30 mm (white) margins from the CTV delineated based on the 4D-CT images (CTV-4D) are displayed on the axial (**a**), coronal (**b**), and sagittal (**c**) planes of planning CT images (AIP CT images of 4D-CT). The OARs of the liver, spinal cord, and kidneys are shown in brown, green and lavender, respectively

All CT images were exported to the treatment planning system (Pinnacle^3^, version 9.10; Phillips Radiation Oncology Systems, Fitchburg, WI) and were registered by the hardware arrangement. The gross tumor volume (GTV) was identified based on the endoscopic examination findings, and it was confirmed that the entire stomach appropriately covered the GTV. The CTV was defined as the entire stomach [19, 27]. The CTV delineated based on the 4D-CT images was defined as the CTV-4D. A PTV was defined as the CTV-4D along with an additional margin, which accounts for intra- and interfractional variations in stomach volume, respiratory movement, and patient set-up error [10, 19]. All patients underwent the treatment with an individually-defined PTV considering age, performance status, and the dose volume histogram, in terms of target coverage and OAR doses. The structure of the OARs was delineated based on the AIP or slow CT images. The PTV structures with 5, 10, 15, 20, 25, and 30 mm margins from the CTV-4D were generated for the retrospective evaluation (Fig. 1). AIP or slow CT images and all structures were exported into the Elekta X-ray volume imaging (XVI) software, version 5.0.4 (Elekta Oncology Systems, Crawley, UK) as references to be used for image guidance.

### 4D-CBCT imaging and target localization method

During the initiation of each actual treatment session, the patient was positioned based on body skin marks and aligned at the isocenter. Before the daily treatment fraction of radiotherapy, 4D-CBCT imaging based on skin marks (skin matching) was performed using the Elekta Symmetry 4D IGRT System (Elekta Oncology Systems, Crawley, UK). The projection data of 4D-CBCT were sorted into 10 respiratory-phase bins. The scan parameters were set to 120 kV, 20 mA, 16 ms per frame, and a slice thickness of 2 mm, with a gantry rotation speed (GRS) of 50° min^−1^ [17, 19]. Automatic registration between planning CT and 4D-CBCT images was performed based on the bone anatomy (bone matching) using the Elekta XVI software. Subsequently, the manual registration between planning CT and 4D-CBCT images was performed based on the stomach anatomy using the axial, coronal, and sagittal images until moving images of the stomach in all 10 phases of the 4D-CBCT images were symmetrically positioned within the PTV structure in the planning CT images (4D soft-tissue matching).

### Evaluation of the required PTV for target localization methods

**Fig. 2:**
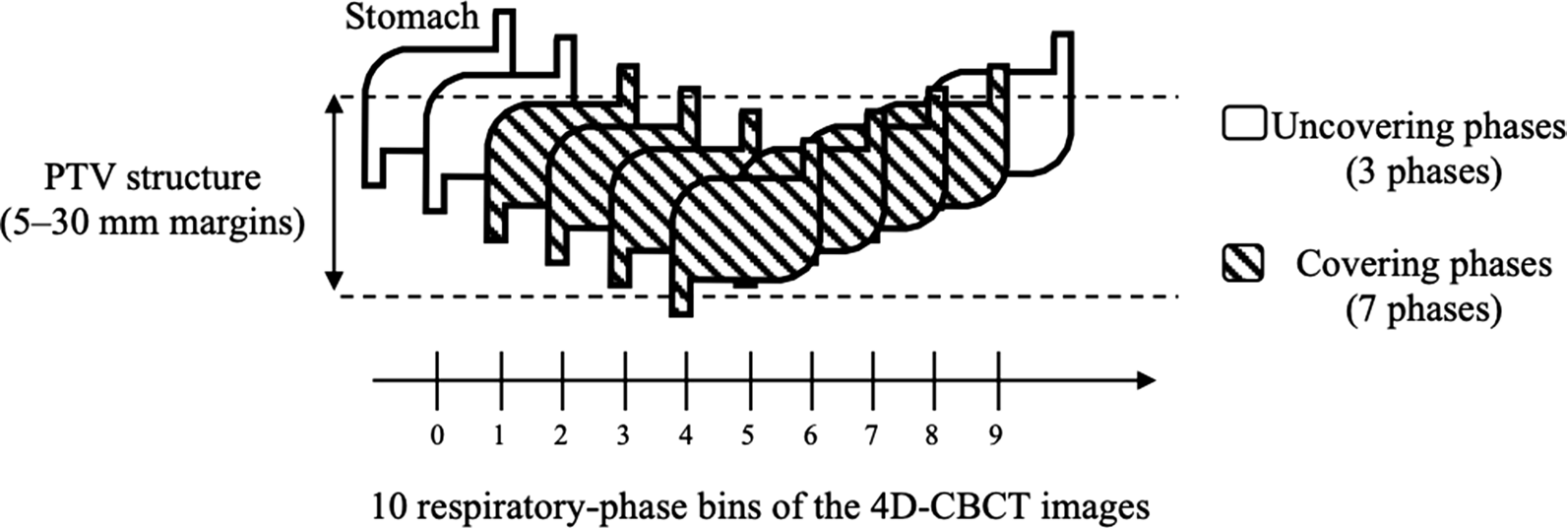
Illustration of how to calculate the covering ratio (CR) of the stomach with PTV structures of 5–30 mm margins, based on the 4D-CBCT images. The CR with PTV structure in the illustration is 70%

We retrospectively evaluated the required PTV to cover the entire stomach, which was confirmed using daily 4D-CBCT images, according to the PTV structures with 5, 10, 15, 20, 25, and 30 mm margins from the CTV-4D. We acquired daily 4D-CBCT images of 10 phases with 15 fractions for each patient (a total of 150 phases per patient). We also compared the required PTV for three target localization methods of the skin, bone, and 4D soft-tissue matchings using daily 4D-CBCT images. The covering phase of the stomach was defined as the phase in which the PTV structures covered the overall stomach and was evaluated by the consensus of two radiotherapists with 4 and 18 years of experience, respectively. For each patient, we calculated the covering ratio (CR) of the stomach with PTV structures of 5–30 mm margins, based on the 4D-CBCT images of a total of 150 phases [CR (%) = (number of stomach covering phases / total of 150 phases) × 100] (Fig. 2) in three target localization methods, and defined a minimum PTV with an average CR of ≥ 95% for all patients as a requirement [19]. A Kruskal–Wallis test was performed to compare the CRs of the three target localization methods. Subsequently, a Dunn–Bonferroni test was performed to compare the CRs of the three methods as a post hoc analysis if the Kruskal–Wallis test result was significant [28]. Statistical significance was defined as a *P* value < 0.05. All statistical calculations were performed using the SPSS software, version 25.0 (SPSS Inc., Chicago, IL, USA).

## Results

### Covering ratio of the stomach with a variable PTV structure

Table 1 presents the average CR of the skin, bone, and 4D soft-tissue matching for eight patients, according to variable PTV structures. The average CR for all patients increased from 17.9 to 100%, 19.6 to 99.8%, and 33.8 to 100%, for skin, bone, and 4D soft-tissue matchings, respectively, as the PTV structures increased from 5 to 30 mm. The CR obtained by 4D soft-tissue matching was significantly superior to that obtained by skin (*P* = 0.013) and bone matching (*P* = 0.008) for a PTV structure of 15 mm.

**Table 1:** Average covering ratios of the stomach based on skin, bone, and 4D soft-tissue matching according to PTV structures of 5, 10, 15, 20, 25, and 30 mm margins for eight patients

PTV structure (mm)	Covering ratio (%)			*P* value
	Skin matching	Bone matching	4D soft-tissue matching	
5	17.9	19.6	33.8	0.323
10	53.8	52.8	76.7	0.053
15	82.5	79.7	98.0	0.003
20	95.2	93.4	99.4	0.186
25	99.2	99.1	100.0	0.320
30	100.0	99.8	100.0	0.368

### Required PTV for target localization methods

**Fig. 3:**
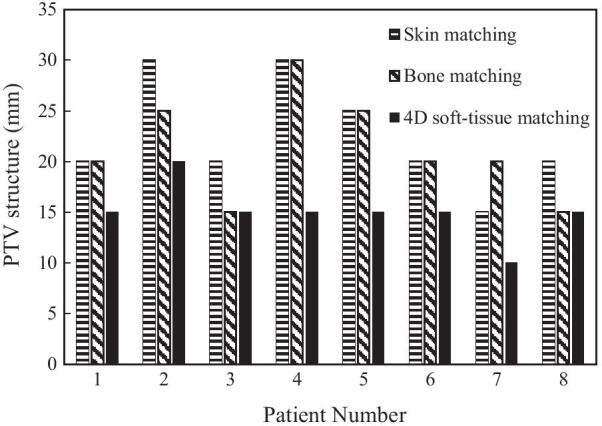
PTV structures with a covering ratio ≥ 95% for skin, bone, and 4D soft-tissue matching for eight patients

**Fig. 4:**
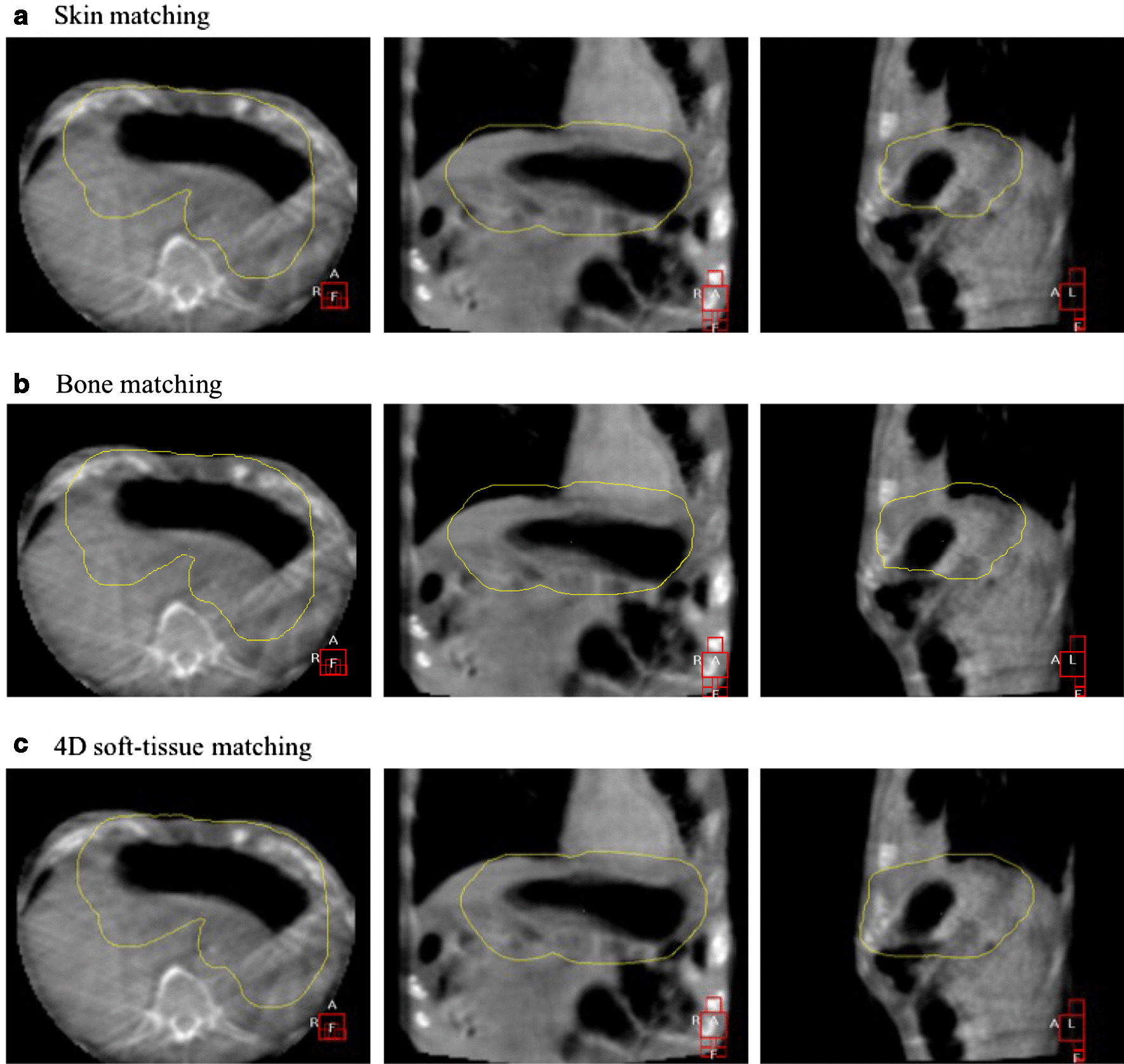
Representative images (patient number 4) of the positional discrepancy between target localization methods based on skin matching (**a**), bone matching (**b**), and 4D soft-tissue matching (**c**) using 4D-CBCT. A PTV structure of 15 mm (yellow) margin from the CTV defined based on the 4D-CT (CTV-4D) is displayed in the 4D-CBCT images. Target localization by skin and bone matching could not cover the entire stomach in the PTV (**a**, **b**). Target localization by 4D soft-tissue matching was able to cover the entire stomach in the PTV (**c**)

Figure 3 shows the PTV structures that yield CR ≥ 95% for skin, bone, and 4D soft-tissue matching for each of the eight patients. The PTV structure that yields CR ≥ 95% for 4D soft-tissue matching is smaller than that of skin matching and is smaller than or equal to that of bone matching. The PTV (average CR ≥ 95%) for all patients required an additional margin of 20 mm (average CR = 95.2%), 25 mm (average CR = 99.1%), and 15 mm (average CR = 98.0%) to CTV-4D for skin, bone, and 4D soft-tissue matchings, respectively (Table 1, Fig. 4).

## Discussion

The results of the current study show that 4D soft-tissue matching provides more precise IGRT with a smaller PTV than skin and bone matching. They also show that a PTV with bone matching is not significantly different from that with skin matching. This indicates that, compared with skin matching, image guidance based on bone matching does not contribute to a reduction of PTV for gastric MALT lymphoma radiotherapy. Methods using 4D soft-tissue matching can be applied not only to assess the daily interfractional variation of the target volume but also to provide precise target localization while reducing the PTV.

In this study, we performed treatment planning using 4D-CT in the FB condition. The use of 4D-CT enables more accurate patient-specific PTV for intrafractional motion caused by respiration [10]. The PTV for gastric MALT lymphoma radiotherapy should consider not only set-up variations but also the interfractional stomach variation. The results of the PTV in the current study reflect the interfractional variation of the stomach volume resulting from using daily 4D-CBCT-IGRT. Based on these results, the CTV-4D along with an additional margin of 15 mm was sufficient to cover the entire stomach only if 4D-CT planning and 4D soft-tissue matching using 4D-CBCT were performed, but it was insufficient for skin and bone matching. Johnson et al. investigated an additional margin required to encompass 95% of the stomach volume using daily megavoltage CT in gastric lymphoma radiotherapy of three patients in the FB condition [8]. They showed that a uniform margin of 22 mm was required with bone matching. The results of the current study based on bone matching are consistent with their results. Moreover, the International Lymphomas Radiation Oncology Group (ILROG) guidelines for treatment planning of gastric lymphoma recommends adding a margin of at least 10 to 20 mm to CTV to accommodate stomach movement [27]. The results of the current study based on 4D soft-tissue matching are consistent with ILROG guidelines. However, a isotropic margin of the current study was not evaluated in three dimensions. The range of the stomach movement was complex and different in the cranio-caudal, right-left, and anterior–posterior directions [6–8, 11]. Therefore, the optimal PTV should be determined in three dimensions. Herein, although the optimal PTV could not be determined, 4D-CT planning and 4D soft-tissue matching using 4D-CBCT were deployed to individualize the PTV considering the interfractional variation in stomach volume [11].

A limitation of our study is the relatively small number of patients. Moreover, we could not evaluate intrafractional changes in stomach volume and respiratory movement during treatment. In-treatment 4D-CBCT could be used to evaluate the intrafractional stomach changes [29]. Based on the results of our study, we recommends using the daily 4D-CBCT. An optimal imaging protocol to balance the image quality with patient exposure to X-rays is under consideration [14, 16].

## Conclusions

In this study, we retrospectively evaluated whether the use of 4D-CBCT reduced PTV by determining the required PTV for target localization methods of the skin, bone, and 4D soft-tissue matching for gastric MALT lymphoma radiotherapy. 4D soft-tissue matching using 4D-CBCT provides a smaller PTV than skin and bone matching. Furthermore, it was found that image guidance with bone matching does not contribute to a reduction of PTV compared with skin matching. This study demonstrates the efficacy of 4D soft-tissue matching using 4D-CBCT for gastric MALT lymphoma radiotherapy.

## Data Availability

The data that support the findings of this study are available from the corresponding author, but restrictions apply to the availability of these data, which were used under license for the current study, and so are not publicly available. However, the authors can provide the licensed data upon reasonable request and with the permission of the Institutional Research Ethics Board of Kumamoto University Hospital.

## Abbreviations

AIP: Average intensity projection
CBCT: Cone-beam computed tomography
CR: Covering ratio
CT: Computed tomography
CTV: Clinical target volume
FB: Free breathing
4D: Four-dimensional
GRS: Gantry rotation speed
GTV: Gross tumor volume
IGRT: Image-guided radiotherapy
ILROG: International Lymphomas Radiation Oncology Group
IMRT: Intensity-modulated radiation therapy
MALT: Mucosa-associated lymphoid tissue
OARs: Organs at risk
PTV: Planning target volume
XVI: X-ray volume imaging
